# The scaffolding protein GRASP/Tamalin directly binds to Dock180 as well as to cytohesins facilitating GTPase crosstalk in epithelial cell migration

**DOI:** 10.1186/1471-2121-14-9

**Published:** 2013-02-26

**Authors:** Myriam A Attar, Lorraine C Santy

**Affiliations:** 1 Department of Biochemistry and Molecular Biology, The Pennsylvania State University, 208 Althouse Lab, University Park, PA 16802, USA

**Keywords:** Cytohesin, GRASP, Tamalin, Dock180, Arf6 and Rac1

## Abstract

**Background:**

The transition of epithelial cells from their normal non-motile state to a motile one requires the coordinated action of a number of small GTPases. We have previously shown that epithelial cell migration is stimulated by the coordinated activation of Arf and Rac GTPases. This crosstalk depends upon the assembly of a multi-protein complex that contains the Arf-activating protein cytohesin 2/ARNO and the Rac activating protein Dock180. Two scaffolding proteins that bind directly to cytohesin 2 organize this complex.

**Results:**

We now have found that Rac activation in response to hepatocyte growth factor (HGF) requires cytohesin 2 and Dock180. GRASP/Tamalin is one of the scaffolds that builds the complex containing cytohesin 2 and Dock180. We determine here that the Ala/Pro rich region of GRASP directly interacts with the SH3 domain of Dock180. By binding to both cytohesin 2/ARNO and Dock180, GRASP bridges the guanine nucleotide exchange factors (GEFs) that activate Arf and Rac, thereby promoting Arf-to-Rac signaling. Furthermore, we find that knockdown of GRASP impairs hepatocyte growth factor (HGF)-stimulated Rac activation and HGF-stimulated epithelial migration.

**Conclusions:**

GRASP binds directly both cytohesin 2 and Dock180 to coordinate their activities, and by doing so promotes crosstalk between Arf and Rac.

## Background

Epithelial cells form barriers that can selectively regulate transport between different compartments. An extensive network of junctions joins the cells into sheets and limits their mobility under normal circumstances. However these cells do become migratory under both normal and pathological conditions. Epithelial cells must migrate during normal development and during the repair of damage. In addition cancerous epithelial cells aberrantly activate pro-migratory pathways during metastasis. Epithelial migration involves a remodeling of the cell’s structure and behavior that starts by redirecting polarity in the direction of migration. At the leading edge, actin rich protrusions and new cell-matrix adhesions anchor the cell to help propel the cell forward and the trailing edge retracts [1]. Epithelial cells can adopt several different types of migration depending on the biological circumstances at hand [2]. During tissue morphogenesis, development and wound healing, epithelial cells move in sheets. In this case, they maintain their cell-cell junctions [3]. Epithelial cells can also detach from each other and migrate individually during development or cancer metastasis [4].

Epithelial cell motility is initiated by various growth factors, such as HGF, EGF, PDGF, VEGF, CSF-1, FGF and TGF-β [5-10]. HGF, also known as Scatter Factor (SF), is a potent motogen for numerous epithelial cells expressing the c-Met receptor [11]. It induces scattering of multiple epithelial cell lines in 2D culture [12-14]. When epithelial cells are grown in 3D cultures, addition of HGF to the growth media initiates tubulogenesis [14,15]. HGF production by mesenchymal cells [16] is increased in the event of injury to epithelia [17]. In addition, HGF is involved in the invasive behaviors of some cancers [18].

A number small GTPases, including members of the Ras, Rho and Arf families regulate the cell shape changes that underlie motility. There are six Arf proteins, and Arf6 in particular has been implicated in the regulation of cell shape and motility. Initially, Arf6 was shown to regulate intracellular trafficking processes like endocytosis and recycling of membrane proteins [19,20]. But it has subsequently been shown that Arf6 is also involved in regulating the actin cytoskeleton during migration and phagocytosis [21-26]. Arf6 is required for HGF stimulated epithelial cell motility [23]. HGF will induce MDCK cells in culture to scatter from islands and increased Arf6 activation is observed as soon as 1 hour post HGF treatment [23,26-28]. More recently, we found that CNK3/IPCEF, a scaffold that binds the Arf-activating cytohesin proteins, is necessary for the activation of Arf6 downstream of HGF and for HGF-stimulated migration [29].

While there are 6 Arf proteins in mammalian cells, a much larger number of proteins have been identified as Arf activating guanosine exchange factors (GEFs). There are 15 identified sec7 Arf GEFs divided into 5 subfamilies. It is thought that the various Arf-GEFs activate Arfs at different subcellular locations and in response to different signals. One class of Arf-GEFs, the cytohesins, has been extensively implicated in the regulation of cell shape and migration. There are 4 cytohesins. Cytohesin1 and 4 are mostly hematopoetic whereas cytohesin 2/ARNO and cytohesin 3/Grp-1 are ubiquitously expressed [30].

Overexpression of cytohesin 2/ARNO enhances cell motility in MDCK cells [26], and the phenotype is strikingly reminiscent of the response of these cells to HGF. Cytohesin 2-induced scattering of MDCK cells requires the activation of Rac1 by the Rac-GEF, Dock180 [31]. Cytohesin 2-dependent Rac activation also depends on the coiled-coil domain in cytohesin 2 [32]. We previously found that cytohesin 2 and Dock180 associate within a larger complex and can be co-immunoprecipitated. IPCEF/CNK3 and GRASP, two scaffold proteins that both bind the coiled-coil domain of cytohesin 2, are necessary for the assembly of this complex, and for cytohesin dependent Rac activation [32]. These data led us to propose a model where one scaffold recruits cytohesin 2 to the membrane in response to upstream signals, while the other acts as a bridge linking cytohesin 2 and Dock180. Our demonstration that CNK3/IPCEF is required for activation of Arf6 by HGF suggests that it is the scaffold that recruits cytohesin 2 in response to upstream signals.

Here, we test the hypothesis that GRASP binds to both Dock180 and cytohesin 2 and bridges the two GEFs. We find that GRASP interacts with Dock180 independently of its ability to bind cytohesin 2. Dock180 and GRASP interact via the SH3 domain of Dock180 and the proline rich domain of GRASP. Furthermore, in addition to physically bridging cytohesin 2 and Dock180, GRASP affects cell migration directly. Knockdown of GRASP inhibits HGF-induced migration in MDCK cells.

## Results

### HGF-induced cell migration and Rac activation are mediated by cytohesins and Dock180

**Figure 1:**
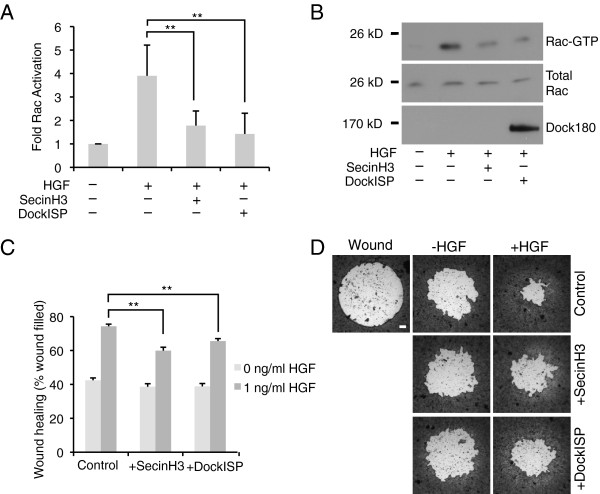
**Inhibition of cytohesin or Dock180 activity impairs HGF-stimulated Rac activation and wound healing. A,B**) HGF-dependent Rac activation is impaired by SecinH3 or dominant negative Dock180. MDCK cells were incubated in the presence or absence of 20 ng/ml HGF for 6 hrs. SecinH3 (15 μM), a specific cytohesin inhibitor or adenovirus encoding the dominant negative Dock180 mutant, DockISP, were added as indicated. Rac-GTP was isolated by pulldown with GST-PBD. Pulldown samples and saved aliquots of the starting lysates were Western blotted with antibodies to Rac and Dock180. Four independent experiments were quantitated by densitometry (A) ** = p < 0.01, paired *T* test. Gels from one of these experiments are shown (**B**). **C,D**) SecinH3 and DockISP inhibit HGF-stimulated wound healing. MDCK cells were plated in 96 well Oris migration chambers (Platypus technologies) around plugs. 24 hours later the plugs were removed to create a wound. Wounded monolayers were incubated in the presence or absence of 1 ng/ml HGF with the addition of 30 μM SecinH3 or adenovirus encoding DockISP as indicated for 18 hours. Cells were then fixed and stained with crystal violet. The area of the remaining wound was measured using ImageJ. C) The extent of wound healing in multiple replicate samples from multiple independent experiments was quantitated: Control (n = 67 wells from 12 experiments); SecinH3 (n = 37 wells from 10 experiments); DockISP (n = 24 wells from 4 experiments). Data shown are mean ± standard error. ** = p < 0.01, *T* test. D) Representative images of the starting wound and wounds after 18 hours of migration in the presence or absence of HGF Bar = 500 μM.

Our previous work has shown that cytohesin 2 can promote epithelial migration by stimulating the activation of Arf6 and the subsequent activation of Rac1 [26,31]. Cytohesin 2 induces Rac activation by associating with the Rac-GEF Dock180. This interaction is mediated by the scaffolding proteins CNK3 and GRASP, and knockdown of GRASP or CNK3 inhibits cytohesin 2-induced Rac activation [32]. We have also found that knockdown of CNK3 inhibits HGF-stimulated Arf6 activation and migration [29]. These data suggest that the cytohesin-dependent Arf-to-Rac signaling module may act during HGF-stimulated motility. In order to test this hypothesis we tested the effect of inhibition of cytohesins and Dock180 on HGF-stimulated Rac activation. We incubated MDCK cells with HGF for six hours and added SecinH3, a specific, small-molecule cytohesin inhibitor, or adenovirus encoding a catalytically inactive and dominant negative Dock180 mutant (DockISP) to selected samples. We then evaluated the effect of inhibiting either GEF on Rac activation using a GST-PBD pulldown assay. When the GEF activity of either cytohesins or Dock180 was impaired, Rac activation by HGF was significantly impaired (Figure 1A and 1B). To further confirm that HGF-induced migration is dependent on the GEF activities of cytohesins and Dock180, we evaluated wound healing in the presence of SecinH3 and DockISP. MDCK cells were grown to form a monolayer around plugs in the Platypus migration chambers. The plugs were removed and the cells were allowed to migrate into the wound in the presence or absence of 1ng/ml of HGF. The suppression of cytohesin or Dock180 activity reduced the amount of wound filled in HGF-stimulated samples, but had no significant effect on basal migration (Figure 1C and 1D). Both results are consistent with the conclusion that cytohesin dependent Arf-to-Rac signaling promotes epithelial cell migration downstream of HGF.

### The scaffolding protein GRASP binds independently to both cytohesin 2/ARNO and Dock180

We know that cytohesin dependent Arf-to-Rac activation depends on the assembly of cytohesins and Dock180 and scaffolding proteins into a multiprotein complex [32]. We proposed that one of the scaffolds recruits cytohesin 2/ARNO in response to upstream signals while the other coordinates the association of Dock180 with cytohesin 2/ARNO. We have found that CNK3 plays a crucial role in initiating Arf activation downstream of HGF [29].

**Figure 2:**
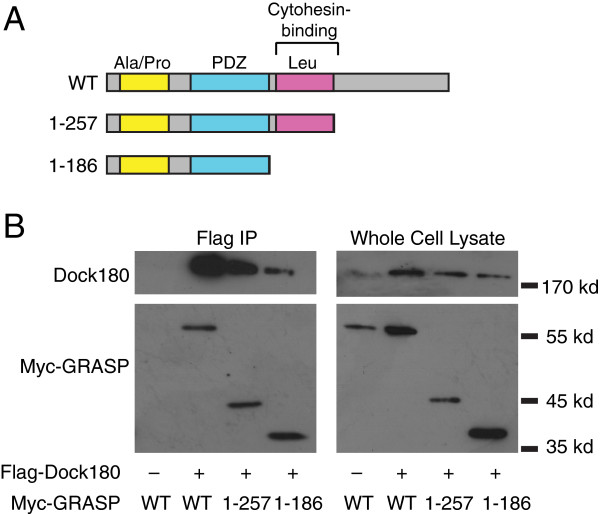
**GRASP co-IPs with Dock180 independently of its cytohesin binding activity. A**) GRASP truncation constructs used in these experiments. All constructs are myc-tagged at the N-terminus. **B**) Hek 293 cells were co-transfected with the indicated GRASP constructs and Dock180. Cells were then lysed, and Dock180 immunoprecipitated with M2-flag resin. The immunoprecipitates were blotted with mouse anti-myc and goat anti-Dock180 antibodies.

GRASP on the other hand, seemed to be a good candidate for bridging Dock180 and cytohesin 2. GRASP, also known as Tamalin, consists of multiple protein-protein interaction domains (Figure 2A), and binds to cytohesin 2/ARNO via its leucine rich region [33-35]. If GRASP acts to links together cytohesin-2 and Dock180, then it should bind directly to both GEFs. GRASP binds to cytohesins via the direct interaction of its leucine rich domain with the cytohesin coiled-coil domain [33]. We have previously shown that Dock180 and GRASP can be co-immunoprecipitated when co-expressed in MDCK cells [32]. Therefore, we produced a series of truncations of GRASP (Figure 2A) in order to narrow down the domain that interacts with Dock180 and to test if GRASP can interact with Dock180 independently of cytohesin 2/ARNO. Cells expressing wild type flag-tagged Dock180 and the various truncations of HA-tagged GRASP were lysed and the post-nuclear supernatant was subjected to immunoprecipitation (as described in Experimental Methods). Dock180 co-immunoprecipitated with GRASP regardless of the presence or absence of its cytohesin-binding domain (Figure 2B). By interacting with both Dock180 and cytohesins via separate domains, GRASP can act as a bridge to co-localize the GEFs for Arf6 and Rac1.

### The SH3 domain of Dock180 and the alanine/proline rich region of GRASP mediate the direct interaction between the two proteins

**Figure 3:**
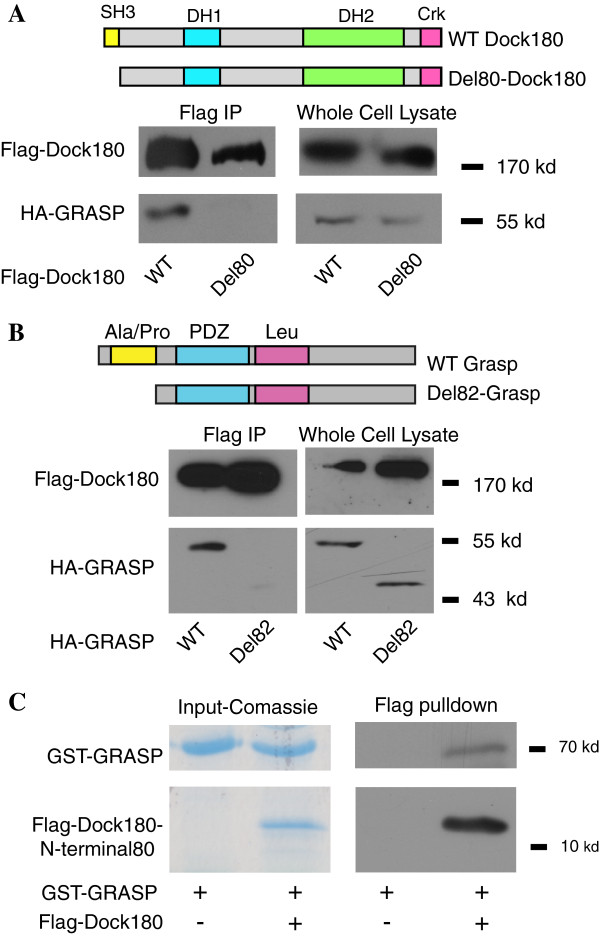
**Dock180 directly binds to GRASP. A**) Localization of the GRASP-binding domain in Dock180. Hek 293 cells expressing HA-GRASP and the indicated VN-flag-Dock180 constructs were lysed, and incubated with M2 anti-flag resin as described in Materials and Methods. The immunoprecipitates as well as saved samples of the starting lysate were blotted with goat anti-Dock180 and mouse anti-HA. **B**) Localization of the Dock180-binding domain in GRASP. Hek 293 cells expressing HA-GRASP or a version lacking the ala/pro rich region (del82-GRASP) and flag-Dock180 constructs were lysed, and incubated with M2 anti-flag resin as described in Experimental Procedures. The immunoprecipitates as well as saved samples of the starting lysate were blotted with goat anti-Dock180 and mouse anti-HA. **C**) GRASP directly binds the SH3 domain of Dock180. E. coli were transformed with pGEX2T-GRASP or pST50Tr-FLAGyGcn5x-Flag-Dock180 (11KDa). The expressed proteins were purified as described in Materials and Methods. GST-GRASP (70 KDa) was eluted from the Sepharose resin using a solution of Glutathione. It was then added to either M2-Flag Sepharose gel or M2-Flag-Dock180 beads. The resin was washed three times and attached proteins were eluted in 30μl sample buffer. GRASP and Dock180 were detected by GelCode (Coomassie) staining and Western blotting with Goat anti-GRASP as well as goat anti-Dock180 antibodies (N-terminal).

We previously demonstrated that the N-terminal 357 amino acids of Dock180 were responsible for the interaction with cytohesin 2/ARNO [32]. Furthermore knockdown of GRASP impairs interaction of Dock180 and cytohesin 2/ARNO as well as cytohesin 2/ARNO induced Rac1 activation. If GRASP is linking cytohesin 2/ARNO to Dock180 then this same region should be required for the interaction of GRASP and Dock180. We found that the N-terminus of Dock180 does indeed mediate the interaction with GRASP (Figure 3A) supporting the hypothesis that GRASP is bridging Dock180 and cytohesin 2/ARNO. Furthermore, we further refined our localization of the region of Dock180 that interacts with GRASP by producing a truncation of the first 80 amino acids of Dock180. This portion of Dock180 encodes a SH3 domain. This Dock180 truncation or full-length Dock180 were transfected along with HA-tagged wild type GRASP into HEK 293 cells. The ability of the Dock180 truncation to interact with GRASP was tested by co-immunoprecipitation. WT GRASP fails to interact with the truncation of Dock180 that lacks the SH3 domain. Similarly we further defined the region of GRASP required for this interaction by producing a truncation of GRASP lacking the N-terminal 82 amino acids, which includes the proline-rich domain. Del82-GRASP did not interact with Dock180 (Figure 3B). Therefore we conclude that the SH3 domain of Dock180 preferentially binds to the proline-rich region of GRASP.

However since these proteins were immunoprecipitated out of cell lysates the interaction could be indirect. In order to determine if the interaction between Dock180 and GRASP is direct, we purified full length GRASP fused to GST and the N-terminal 80 amino acids of Dock180 fused to a Flag tag from E.coli and allowed them to interact in vitro. GRASP interacted with the N terminal 80 amino acids of Dock180 (Figure 3C). This confirms that GRASP can directly interact with Dock180 as well as with cytohesins and can act as the anchoring scaffolding protein that bridges cytohesin 2 and Dock180 and promotes therefore Arf6 to Rac1 activation.

**Figure 4:**
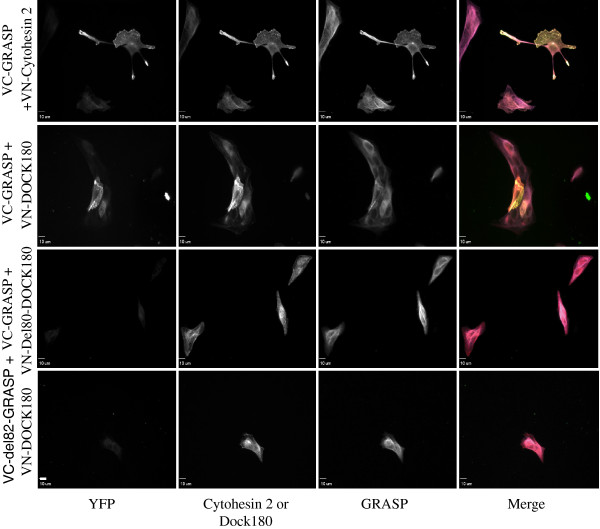
**Dock180 interacts with GRASP in live cells.** A split YFP system was used to demonstrate the interaction of Dock180 and GRASP in cells. Dock180 and GRASP were respectively labeled with the N and C-terminal halves of venus (VN-Dock180 or VC-GRASP). Neither VN nor VC is fluorescent by itself. YFP fluorescence is reconstituted if the two fusion partners interact, thereby bringing VN and VC into close proximity so that a functional YFP can be formed. MDCK cells were transfected with the indicated constructs using Neon® (1400, 20, 2). They were then plated on Fibronectin coated coverslips. After 18 hours, Cells were fixed and stained with mouse anti-myc (cytohesin 2) or mouse anti- Flag (Dock180) followed by Dylight™ 594- conjugated anti-mouse secondary antibody and rabbit anti-HA (GRASP) followed by Dylight™649- conjugated anti-rabbit secondary antibody. In the merge cytohesin 2 or Dock180 are psuedocolored red, GRASP is psuedocolored blue, and the YFP channel detecting Venus indicating that the 2 expressed proteins interact is pseudocolored green. Bar, 10 μm.

The co-immunoprecipitation experiments demonstrate that the Dock180 SH3 domain can interact with GRASP’s proline-rich domain. We wanted to confirm that these proteins interact within intact cells. The Bi-Molecular Fluorescent complementation assay, or BiFC for short, is based on the assembly of the N and C terminal fragments of Venus into a fluorescent protein. The individual fragments VN and VC are not fluorescent individually. The detection of fluorescence depends on in vivo assembly of the fragments into a functional YFP molecule, which only occurs if they are fused to proteins that interact [36]. We designed VN-Dock180 and VC-GRASP and co-transfected them into MDCK cells. Cells were then fixed and stained as described in Experimental Methods. GRASP and cytohesin 2/ARNO are known to interact via the coiled-coil domain of cytohesin 2/ARNO and the leucine rich region in GRASP [33]. We used the interaction of VN-cytohesin 2 and VC-GRASP as a positive control for the detection of YFP (Figure 4 row #1). VN-Dock180 and VC-GRASP also produce YFP fluorescence when co-expressed in MDCK cells (Figure 4 row #2). VN-Del80-Dock180 fails to produce YFP when co-expressed with VC-GRASP (Figure 4 row #3), confirming the co-immunoprecipitation data shown in Figure 3. When MDCK cells were transfected with a plasmid containing the truncation of VC-GRASP lacking the first 82 amino acids of GRASP, and full length VN-Dock180, the two proteins also failed to show interaction by detection of YFP (Figure 4 row #4). These results confirm that the SH3 domain of Dock180, located in the N-terminal 80 amino acids, and the Ala/Pro rich region of GRASP mediate the interaction of the two proteins, thus confirming the observations made in the co-IP studies shown above.

### GRASP knockdown inhibits HGF stimulated migration and Rac activation

**Figure 5:**
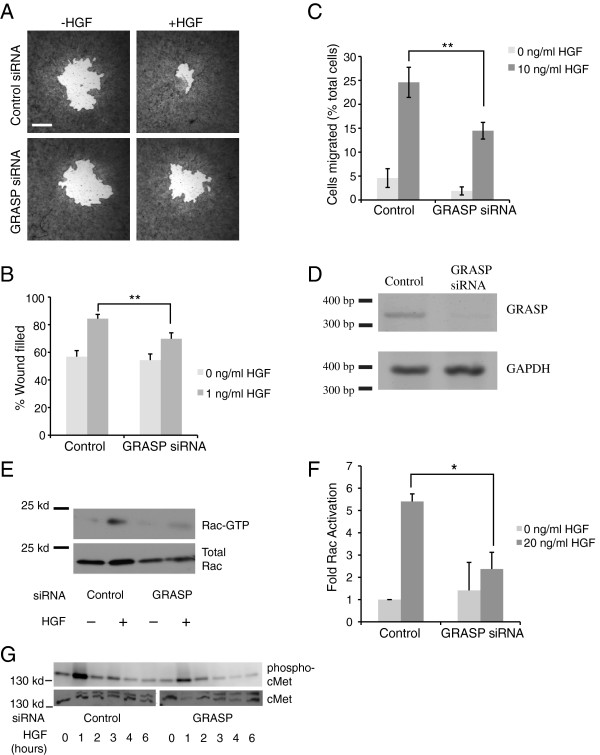
**Knockdown of GRASP inhibits HGF induced migration and Rac activation in MDCK cells. A**) GRASP levels in MDCK cells were reduced by transfection of siRNA and cell migration measured using the Oris migration chamber. **B**) Cell migration was quantitated by measuring the percent of the starting open area covered. Data shown are the mean ± standard error of at least 15 separate samples. **C**) GRASP knockdown and control cells were allowed to migrate in a transwell chamber toward 10 ng/ml HGF. Data shown are the mean ± standard error of at least 15 separate experiments. Migration of the knockdown and control cells in both B. and C was compared using a standard t-test. ** p < 0.01. **D**) MDCK cells were transfected with control or GRASP-targeting siRNA as described in Experimental Procedures. After 48 hours mRNA was isolated and RT-PCR of the canine GRASP or GAPDH performed. **E**) MDCK cells were transfected with siRNAs and 24 hours later were split onto duplicate plates. The next day the cells were incubated in the presence or absence of HGF for 5 hours and active Rac isolated by binding to GST-PBD. Rac levels in saved aliquots of the starting lysates and the isolated Rac-GTP were detected by Western blotting. **F**) Rac activation levels in 4 independent knockdown experiments were normalized to the level of active Rac in the control 0 ng/ml HGF sample. Rac activation in the HGF stimulated control and GRASP knockdown samples were compared using a paired t Test. Data shown are mean ± standard error. * = p < 0.05. **G**) MDCK cells were transfected with control or GRASP siRNAs. After 48 hours the cells were treated with 20 ng/ml HGF for the indicated times, harvested and blotted for phosphorylated cMet and total cMet.

Our data suggest that cytohesin 2/ARNO and Dock180 interact indirectly via GRASP and that HGF stimulated Rac activation and migration require both cytohesins and Dock180. Therefore, we hypothesized that both HGF-induced migration and HGF-stimulated Rac activation would be impaired by knockdown of GRASP. MDCK cells were transfected with control or GRASP targeting siRNAs (Figure 5D). The HGF-stimulated and basal levels of migration of these cells were tested using both a wound healing assay and a transwell migration assay (Figure 5A-C). HGF-induced migration, but not basal migration, is significantly decreased after reducing GRASP expression (Figure 5B,C). We hypothesize that this reduced migration is due reduced activation of Rac in response to HGF. We determined the amount of active Rac in MDCK cells transfected with control or GRASP-targeting siRNAs in the presence and absence of HGF stimulation. The GRASP knockdown cells had reduced levels of HGF-stimulated Rac activation (Figure 5E,F). This reduced level of Rac activation was not due to impaired cMet activation in response to HGF. GRASP knockdown cells had a similar pattern of cMet activation to that seen in control cells with a peak of cMet phosphorylation at 1 hour of HGF treatment (Figure 5G). We conclude that the GRASP mediated association of cytohesins and Dock180 is required for Rac activation and migration in response to HGF.

## Discussion

**Figure 6:**
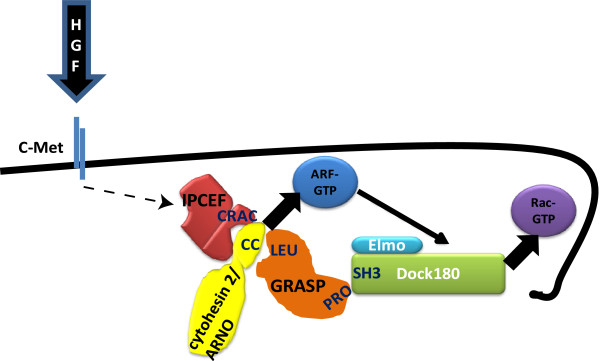
**A comprehensive model for the dual action of GRASP and CNK3/IPCEF in promoting Arf to Rac crosstalk.** GRASP and CNK3/IPCEF play distinct roles in bringing this multiprotein complex together. One of the scaffolds, CNK3/IPCEF recruits cytohesin 2 to the membrane in response to upstream signals (HGF) while the other, GRASP, binds both cytohesin 2 and Dock180 providing a physical bridge. Assembly of this complex promotes HGF-stimulated Rac activation and migration.

In this study we demonstrated that GRASP/Tamalin, in addition to binding cytohesins, also interacts with Dock180. We have previously shown that both GRASP and IPCEF/CNK3 are required for the interaction of cytohesin 2/ARNO and Dock180 and for cytohesin induced Rac activation. The need for two scaffolding proteins to bring about this pro-migratory protein complex was surprising. We hypothesized that one scaffold recruits cytohesin 2/ARNO in response to upstream signals, while the other bridges cytohesin 2/ARNO and Dock180. Further studies in our lab showed that the CNK3/IPCEF acts downstream of HGF and c-met to activate Arf6 [29]. Therefore, we hypothesized that GRASP is the scaffolding protein that brings the GEFs of Arf6 and Rac1 together by binding to both. We have shown here that GRASP binds directly to Dock180, independently of its cytohesin binding domain. These data provide evidence to support the model that GRASP acts to bridge Dock180 and cytohesin 2/ARNO. Truncations of Dock180 and GRASP allowed us to determine that the SH3 domain at the N-terminus of Dock180 and the proline rich region at the N-terminus of GRASP are the regions that interact. Furthermore, knockdown of GRASP impairs HGF-stimulated migration. These studies identify GRASP as a lynchpin molecule that links cytohesin-dependent Arf activation to Dock180 dependent Rac activation (Figure 6).

In addition to meditating protein-protein interactions, the SH3 domain present in some Dock180 related proteins also regulates the GEF activity of these proteins. In fact, the SH3 domain has autoinhibitory properties [37]. A Dock180 mutant that lacks the SH3 domain activates Rac1.5 fold higher than the wild type Dock180. Interaction of this domain with GRASP might have the further effect of relieving this auto-inhibition.

Dock-related proteins are atypical GEFs for Rho GTPases. They differ from the bigger class of Rho GEFs known as the (DH-PH)-containing family of GEFs [38-40]. There are 11 mammalian Dock-related proteins divided into 4 subfamilies [41]. The DOCK-A and DOCK-B subfamilies contain a SH3 domain at their N-termini that differentiates them from the DOCK-C and DOCK-D subfamilies. The presence of a SH3 domain suggests that other members of the DOCK-A and DOCK-B families may be able to bind GRASP and coordinate with cytohesin dependent Arf activation. The members of these sub-families include Dock180, Dock2 and Dock5 for the A subfamily and Dock3 and 4 for the B subfamily.

Dock2 is present primarily in hematopoetic cells, whereas Dock180 is absent in these cells. Dock2 plays a role in lymphocyte development, homing, activation, adhesion, polarization and migration [41-44]. There are hematopoetic isoforms of cytohesins, namely Cytohesin 1 and Cytohesin 4. Cytohesins can regulate integrin function and trafficking and therefore migration and adhesion in epithelial cells [26,45,46] and in hematopoetic cells [47,48]. No studies have yet investigated coordination between hematopoetic Dock-related proteins and cytohesins, so it remains to be seen if similar mechanisms are at work in these cells.

Dock3 in conjunction with NEDD9 promotes EMT, mesenchymal migration and metastasis of cancer cells [49,50]. Mesenchymal migration is characterized by the activation of Rac and the production of lamellipodia. This type of migration is consistent with HGF and cytohesin-induced motility. Furthermore activation of Arf6 has also been correlated with enhanced metastasis [27,51]. Similarly to the effects of Dock2 on hematopoetic cell migration, it is not yet known if cytohesins regulate NEDD9/Dock3 stimulated migration.

Another area where both DOCK-A or DOCK-B proteins and cytohesins have been implicated is the neuronal development. Dock3 regulates neurite outgrowth [52,53], while Dock 4 has been implicated in the regulation of dendritic development [54], Rac-dependent cell migration [55] and tumorigenesis [56]. Cytohesins have also been shown to regulate neurite outgrowth and dendritic branching in hippocampal neuron cultures [57-59]. All of these functions involve cytohesins and the SH3-containing DOCK family members, and therefore might involve coordination by GRASP.

## Conclusions

The results reported here in conjunction with our earlier studies allow us to build a detailed model to explain how cytohesins coordinate with Dock180 to promote Rac activation and cell migration as shown in Figure 5. We propose that Arf6 induced Rac1 activation depends on the assembly of a multiprotein complex containing the GEFs, cytohesin 2 and Dock180/Elmo1, as well as the necessary scaffolding proteins that bring the complex together and to its proper location in the cell (Figure 6).

An external stimulus, in this case HGF, activates a cascade of events. This leads to the recruitment of cytohesin 2/ARNO where it’s needed for the activation of Arf6 [29]. The proper functioning of cytohesin 2/ARNO is not only dependent on its catalytic ability to facilitate the GDP to GTP exchange on Arfs, but also on its interactions with scaffolding proteins [32]. We demonstrated that CNK3/IPCEF is the scaffolding protein necessary for activation of Arf6 [29]. However, cytohesin 2 also binds another scaffolding protein, GRASP/Tamalin [33]. And as we previously described, knockdown of the expression of either CNK3/IPCEF or GRASP/Tamalin impairs the assembly and function the multiprotein complex described above and the ability of cytohesin 2/ARNO to stimulate Rac [32]. The data shown in this paper defines the role of GRASP as providing a physical bridge between cytohesin 2 and Dock180, thus is coordinating the activation of Arf6 and Rac1.

The scaffolding protein GRASP completes the picture on the large multiprotein complex that brings Arf6 and Rac1 together. The proline rich region of GRASP binds the SH3 domain of Dock180, while its leucine rich domain binds cytohesin 2. GRASP therefore helps GEFs co-localize activation of GTPases, and promotes crosstalk between Arf and Rac.

## Methods

### Antibodies and reagents

The mouse anti-HA (16B12) was purchased from Covance (Princeton, NJ). Goat anti-Dock180 antibodies (C-19, N-19) were obtained from Santa Cruz Biotechnology (Santa Cruz, CA). The mouse anti-Flag antibody was obtained from Sigma (St Louis, MO). DyLight™ 649 conjugated donkey anti-rabbit as well as DyLight™ 594 conjugated donkey anti mouse were purchased from Jackson ImmunoResearch laboratories (West Grove, PA). Anti phospho-cMet (Y1234/1235 clone D26) and Anti-cMet (clone 25H2) were purchased from Cell Sigaling (Danvers, MA). M2 anti-flag resin was purchased from Sigma (St. Louis, MO). And CL4B beads were obtained from Fluka (Germany) and GelCode Blue and glutathione-sepharose from Thermo Scientific (Rockford, IL). Western blots were processed by incubation with HRP-labeled secondary antibodies followed by Millipore Immobilion ECL reagent. Blots were developed using X-Ray film or with the c-Digit imaging system (LI-COR Biosciences, Lincoln, NE).

### Cell lines

Tet-off MDCK cells were obtained from Clontech. 293H cells were obtained from Invitrogen (Carlsbad, CA). The T23 line of MDCK II as well as the 293H cells were maintained in DMEM supplemented with 10% FBS and penicillin, streptomycin and fungizone. All cells were maintained at 37°C and 5% CO_2_. Cell media was purchased from Mediatech (Manassas, VA) and FBS from Gemini (West Sacramento, CA).

### siRNA mediated knockdown

The siRNA targeting human and dog GRASP (target sequence: GCTTTGAGATCCAGACTTA) was obtained from Dharmacon (Lafayette, CO) and BlockIt® control siRNA from Invitrogen (Carlsbad, CA). siRNAs were transfected into T23 cells using the Neon® system from Invitrogen (Carlsbad, CA). Transfections were carried out using the manufacturer’s suggested protocol: (1650 mV, 20 ms, 1 pulse) for MDCK cells.

### Migration assay

Control or GRASP-targeting siRNAs were transfected into the cells using Neon. Cells (7×10^5^) were seeded in the wells of the Oris migration chambers (Platypus technologies, Madison, WI) and incubated for 24 hours. Plugs were removed and media was refreshed before addition of HGF (1 ng/ml). Cells were allowed to migrate for another 18 hours. Cells were fixed with 4% paraformaldehyde and stained with a 0.1% solution of crystal violet. Images of the chambers were taken and cell migration was quantified by comparing the empty areas in the control versus GRASP knockdown on Image J. For the transwell migration assay, GRASP knockdown was performed as outlined above. The cells were allowed to migrate through the chambers as described in Attar et. al., 2012 [29].

### Primers

Del82-GRASP forward: 5^′^-CAC GCTCGAGACCATGGCATACCCATACGATG TCCTGACTATGCAGGCTCAGGATTCCGTTG-3^′^ and Del80-Dock180: 5^′^-CGCGCGGCCGCATGGACTACAAAGACGATGAAGATAAAGTCATCCCGGGTGACCTCCCCC-3^′^.

### Immunoprecipitation

HEK 293 cells were transfected (calcium phosphate) with plasmids encoding various forms of Dock180 and GRASP. They were allowed to express overnight. Cells were lysed in 50mM Tris, pH 7.5, 150mM NaCl, 10 mM NaF, 1 mM NaVO_4_, 10 mM sodium pyrophosphate, 1% Triton X-100 and 0.1 mM PMSF and 1 mg/ml each pepstatin, leupeptin, and antipain. Lysates were clarified with Sepharose CL-4B beads and unclarified materials removed after 10 minutes at 12,000 × g. Some of the cleared cell lysate was saved (3% was used for protein expression) and the remainder was then incubated rotating overnight 4°C with acid-washed M2-anti Flag resin. The process involved washing the resin three times with 1X TBS followed by 2 washes with 0.1M glycine pH 3.5 and finished with 3 more washes with TBS. IPs were washed three times with lysis buffer and once with TBS. Precipitated proteins were eluted into SDS-PAGE sample buffer and the samples were boiled for 3 minutes then analyzed by Western blot.

### cMet activation

MDCK cells were transfected with siRNAs as described for migration assays. 48 hours later the cells were treated with 20 ng/ml HGF for the indicated times and harvested as described for immunoprecipitations. Unsolubilized material was removed by centrifugation and the supernatant Western blotted with anti-phospho-cMet and anti-cMet antibodies.

### Immunofluorescence

MDCK were plated on fibronectin coated glass coverslips (40 μg/ml). They were fixed and stained as previously described [26]. Cells were observed and photographed using an Olympus IX81 equipped with SlideBook5 software for image processing.

### RT-PCR

Total RNA was isolated using the RNeasy kit (Qiagen). Custom primers to amplify bases 153–511 of canine GRASP, and ReadyMade primers to amplify GAPDH were obtained from Integrated DNA Technologies. RT-PCR was performed with 0.5 μg total RNA as template for GAPDH and 2 μg as template for GRASP using the Qiagen One-Step RT-PCR kit.

### In Vitro binding assay

The coding sequence of GRASP was inserted to the pGEX2T plasmid to produce an in-frame fusion of GRASP to GST. This plasmid was transfected into BL21 E. coli. Cultures were grown to OD600 = 0.4 and then expression of GST-GRASP was induced by addition of IPTG for 4 hours. Cells were then lysed in PBS + 0.01% Triton X-100 for 10 minutes. The lysate was cleared by centrifugation and added to Glutathione-Sepharose beads. After rotation for 2 hours, beads were washed and GST-GRASP was eluted using a 10 mM solution of glutathione.

The coding sequence of the N-terminal 80 amino acids of Dock180 was fused in frame to a Flag tag using the bacterial expression plasmid pST50 [60] and expression was induced as indicated above. Cells were lysed in 50 mM tris pH 7.5, 20% sucrose and 10% glycerol. The cleared lysate was added to M2-Flag gel and incubated rotating for 2 hours then washed with lysis buffer to remove unbound proteins.

Eluted GST-GRASP was added to either the Dock180 conjugated M2-Flag resin or to just the M2-Flag gel as a control. TBS was added and samples were rotated at 4°C for 2 hours washed and the proteins were then eluted in SDS-PAGE sample buffer. Proteins were detected by GelCode staining and Western blot using the goat anti-GRASP and the N-term goat anti-Dock180 antibodies.

## Abbreviations

ARNO: Arf nucleotide binding site opener; CNK3: Connector enhancer of KSR 3; CSF-1: Colony stimulating factor 1; DH-PH: Dbl homology–pleckstrin homology; EGF: Epithelial growth factor; FGF: Fibroblast growth factor; GAP: GTPase activating protein; GAPDH: Glyceraldehyde 3-phosphate dehydrogenase; GEF: Guanine nucleotide exchange factor; GRASP: Grp1 associated scaffold protein; Grp1: General receptor for phosphoinositides-1; HGF: Hepatocyte growth factor; IPCEF: Interacting protein for cytohesin exchange factor; MDCK: Madin-Darby canine kidney; NEDD9: Neural precursor cell expressed developmentally down-regulated protein 9; PBS: Phosphate buffered saline; PDGF: Platelet derived growth factor; SF: Scatter factor; SH3: Src homology 3; TGF-β: Transforming growth factor beta; VEGF: Vascular endothelial growth factor.

## Competing interests

The authors declare that they have no competing interests.

## Authors’ contributions

MAA and LCS performed the experiments. LCS conceived the study and contributed to its design. MAA drafted the initial manuscript. MAA and LCS edited the manuscript. All authors have read and approved the final manuscript.
